# Establishing a TaqMan qPCR method for identification and quantification of *Fritillaria hupehensis* as a common adulterant of *Fritillaria thunbergii* Miq

**DOI:** 10.3389/fphar.2026.1758900

**Published:** 2026-05-12

**Authors:** Cuiping Yan, Jiabing Xu, Rong Qu, Shuchen Duan, Hui Ye, Feng Wei, Wenjuan Zhang

**Affiliations:** 1 Taizhou Institute for Drug Control, Taizhou, China; 2 School of Traditional Chinese Pharmacy, China Pharmaceutical University, Nanjing, China; 3 National Institutes for Food and Drug Control, Beijing, China

**Keywords:** adulteration, *Fritillaria hupehensis*, *Fritillaria thunbergii* Miq., identification, quantification, TaqMan real-time qPCR

## Abstract

**Background:**

*Fritillaria hupehensis* (HBBM) is frequently found as an adulterant in *Fritillaria thunbergii* Miq. (ZBM) owing to its similar appearance and lower price, resulting in risk of unstable medicinal effects of ZBM.

**Methods:**

A highly specific TaqMan real-time quantitative polymerase chain reaction (qPCR) method was established to identify HBBM from ZBM based on the 47 bp amplicon located on the internal transcribed spacer region of the nuclear ribosomal DNA.

**Results:**

The qPCR assay developed herein could specifically distinguish ZBM from HBBM. The sensitivity study showed that the detectable DNA template concentration of HBBM for this assay was 0.001 ng/μL. The efficiency of the optimized qPCR assay was evaluated to be 90.2% (R^2^ = 0.9990, slope = −3.581). A standard-curve-based quantification method was established to determine the proportion of HBBM adulteration in a binary mixture with ZBM, and the equation of the standard curve was determined as y = −3.192x + 18.316 with R^2^ = 0.9949. This quantification approach was evaluated using the ΔCt parameter (difference in Ct values between sample and reference standard); furthermore, the test accuracy was calculated using the recovery rate that ranged from 81.60% to 113.85% for mixed powder samples containing different proportions of HBBM (1%, 5%, 25%, 50%, and 100%). The short amplicon of length 47 bp ensured efficient detection and accurate quantification of dried decoction pieces.

**Conclusion:**

The TaqMan real-time qPCR assay developed in this study has high efficiency, sensitivity, and specificity; this method is applicable for not only authentication of ZBM and HBBM but also quantitative detection of a binary mixture or adulterated botanical drugs. Furthermore, it provides a reference for qualitative control of other traditional Chinese medicines.

## 1 Introduction

*Fritillaria thunbergii* Miq. and *Fritillaria hupehensis* Hsiao et K.C.Hsia are both officially recorded in the Pharmacopoeia of the People’s Republic of China (2020). Their dried bulbs are commonly known as Zhe Beimu (ZBM) and Hubei Beimu (HBBM), respectively. As members of the Liliaceae family, both plants are widely used in traditional Chinese medicine (TCM) for the treatment of cough and other respiratory diseases. ZBM was first documented in Shennong Materia Medica and is one of the most commonly used herbal medicines in China (Nile et al., 2021; Wang et al., 2021); it is mainly produced in the southeastern coastal and south-central regions, particularly in Zhejiang and Jiangsu provinces, and is frequently included in classic formulas such as Ermu Ningsou Wan, Juhong Wan, Neixiao Luoli Wan, and Danggui Beimu Kushen Wan (Li et al., 2019). In contrast, HBBM is primarily cultivated in Hubei, Chongqing, Hunan, and surrounding areas; its therapeutic emphasis properties include clearing heat, transforming phlegm, relieving cough, and alleviating distension, which make it particularly suitable for treating lung heat and phlegm-heat coughs in TCM (Zhang et al., 2007).

**FIGURE 1:**
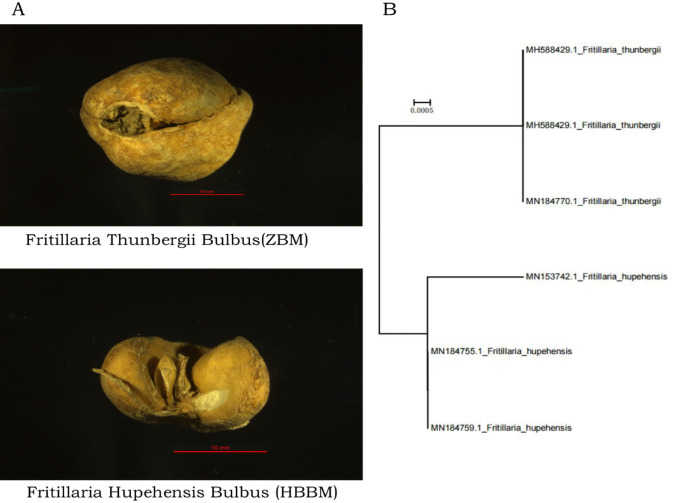
**(A)** The photoes of ZBM and HHBM; **(B)** Phylogenetic tree generated from the downloaded ITS sequences.

ZBM and HBBM have been widely used as TCMs for over 2000 years (Guo et al., 2021; Zhang et al., 2008); both plants share a close phylogenetic relationship, which has resulted in highly similar morphological characteristics and chemical substances, particularly in their bulbs. The bulbs of both plants are small, off-white in color, almost spherical, and include many bulbils (Figure 1A). Owing to certain differences in their metabolites and pharmacological effects, it is essential to accurately distinguish between these two botanical drugs to ensure clinical safety and efficacy. Currently, the ZBM available in the market is mixed or adulterated with large amounts of HBBM, particularly after being processed into decoction pieces and preparations. The older identification methods for ZBM and HBBM primarily rely on traditional approaches, such as morphological, microscopic, and sensory identification. However, these methods are largely dependent on experience and yield results with poor reliability, which can no longer meet the current demands for TCM authentication. Hence, modern physicochemical techniques, including thin-layer chromatography (TLC), high-performance liquid chromatography (HPLC), gas chromatography (GC), and mass spectrometry (MS), have been widely employed to qualitatively and quantitatively analyze the tissue structures, ergastic substances, and indicative components in TCMs for quality control and authenticity purposes (Gao et al., 2019; Li et al., 2020; Sun et al., 2009). Nevertheless, ZBM and HBBM are highly similar in appearance and chemical composition. Studies have shown that both plants contain comparable alkaloid components, such as peimine and peimisine, which cannot be effectively distinguished by TLC or HPLC alone. To overcome these limitations, molecular-marker-based methods have been developed. Studies exploring the molecular markers and genomic characteristics have revealed that the *matK* and *rps16* genes contain sufficient molecular markers to distinguish ZBM from HBBM; by leveraging these genetic traits, it is possible to identify ZBM and other *Fritillaria* species. However, this approach is limited to qualitative identification and cannot quantitatively detect adulteration (Moon et al., 2018a). In the context of botanical drug authentication, quantitative detection has great practical significance as it enables not only the identification of adulterants but also accurate assessment of the adulteration levels, providing essential support for quality and clinical safety evaluations. Therefore, it is necessary to establish a quantitative polymerase chain reaction (qPCR) method for differentiating and identifying these two herbs effectively.

The TaqMan probe real-time fluorescent qPCR has become an important and widely used tool in food quality control, microbial analyses, clinical diagnoses, and other biomedical fields because of its obvious advantages, such as reliable specificity, higher sensitivity, accurate quantification, good reproducibility, simple operation, and faster detection compared to other methods (Garrido-Maestu et al., 2018; Spanoghe et al., 2017; Tichy et al., 2020; Wu et al., 2017). It is particularly suitable for both detecting and quantifying specific targets in samples containing multiple species. However, reports on its application to quality control of TCMs have been very limited till date and have been mainly focused on qualitative detection (Han et al., 2016; Moon et al., 2018b; Villa et al., 2017; Wang et al., 2018; Zhang et al., 2019). In the present study, we developed a TaqMan qPCR method to specifically identify and accurately quantify HBBM in a mixture with ZBM. Considering the fragmented nature of DNA in processed botanical drugs, a short-amplicon strategy was employed to enhance the reliability of detection from decoction pieces while providing a feasible tool to evaluate the authenticity of botanical drugs.

## 2 Materials and methods

### 2.1 Instruments and reagents

The devices used in this study were as follows: real-time fluorescent qPCR (Bio-Rad CFX96 Touch); analytical balance (Mettler XS204); ultrapure water system (Millipore); ball mill (MM400, Retsch); NanoDrop One ultra-micro spectrophotometer (Thermo Fisher Scientific); desktop high-speed freezing centrifuge (Eppendorf Centrifuge 5427 R); Qubit 4 fluorometer (Thermo Fisher Scientific); fully automatic capillary electrophoresis nucleic acid analyzer (Qsep100, BIOptic). For the analyses, we used DNeasy mericon Food Kit (cat. no. 69514, QIAGEN), QIAquick® spin columns (cat. no. 1112323, QIAGEN), spin columns (CapitalBio Technology), probe qPCR mix (2×, cat. no. RR392, TaKaRa), and sterilized distilled water. The probes and primers used in the study were synthesized by GENEWIZ (Azenta Life Sciences).

### 2.2 Sample collection and identification

The botanical drugs *F. thunbergii* Miq. (Liliaceae; *F. thunbergii* Miq. dried bulb) and *F. hupehensis* (Liliaceae; *F. hupehensis* dried bulb) were procured from a planting base, while their decoction pieces were obtained from Bozhou medicinal materials market. Then, the fresh bulbs were washed, thoroughly moistened, cut into thick slices or broken into fragments, and dried. All samples were authenticated by Prof. Ye Hui from Taizhou Institute for Drug Control. The voucher specimens of all medicinal materials are deposited in the Herbarium of the Traditional Chinese Medicine Resource Center, Taizhou Institute for Drug Control. A total of 15 batches of samples were collected, and two batches of standard reference were purchased from the National Institutes for Food and Drug Control (NIFDC) (Table 1). The representative samples of ZBM and HBBM were collected from their main production areas covering different geographical regions and processing types to support the development and validation of a species-specific quantitative detection method.

**TABLE 1:** Information regarding the samples used in this study.

Species	Sample no.	Origin	Batch no.	Location	GPS coordinates (decimal degrees)
*Fritillaria thunbergii* Miq.	ZBM-1	Planting base	240526	Haian, Taizhou	32.53° N, 120.47° E
	ZBM-2		240527	Jinjiang, Taizhou	32.009° N, 120.263° E
	ZBM-3		240527	Haimen, Taizhou	28.67338° N, 121.44034° E
	ZBM-4		240529	Dongtai, Taizhou	32.8523° N, 120.3095° E
	ZBM-5	Market	240301	Pan’an, Zhejiang	29.05368° N, 120.43758° E
	ZBM-6		231201	Pan’an, Zhejiang	29.05368° N, 120.43758° E
	ZBM-7		240229	Pan’an, Zhejiang	29.05368° N, 120.43758° E
	ZBM-8		24040902	Pan’an, Zhejiang	29.05368° N, 120.43758° E
	ZBM-9		240102801	Pan’an, Zhejiang	29.05368° N, 120.43758° E
	ZBM-10		240118001	Pan’an, Zhejiang	29.05368° N, 120.43758° E
	ZBM-SD	Standard reference from NIFDC	120972–201906	Pan’an, Zhejiang	29.05368° N, 120.43758° E
*Fritillaria hupehensis*	HBBM-1	Market	240301	En’shi, Hubei	30.30000° N, 109.48333° E
	HBBM-2		240225	En’shi, Hubei	30.30000° N, 109.48333° E
	HBBM-3		2403006	En’shi, Hubei	30.30000° N, 109.48333° E
	HBBM-4		240313	En’shi, Hubei	30.30000° N, 109.48333° E
	HBBM-5		240403	En’shi, Hubei	30.30000° N, 109.48333° E
	HBBM-SD	Standard reference from NIFDC	120962–202106	En’shi, Hubei	30.30000° N, 109.48333° E

### 2.3 DNA extraction

Before DNA extraction, all bulbs were washed with 70% ethanol and distilled water to eliminate any potential PCR inhibitors. After washing and drying, the bulbs were ground into a fine powder at low temperature. Approximately 20 mg of this powder was then used to extract the sample DNA. The DNAsecure novel plant genome DNA extraction kit (lot no. A0308A, Tiangen, Beijing, China) was used for the genomic DNA extraction, and the purified DNA was dissolved in 50 μL of sterile double-distilled H_2_O. The concentration and purity of the genomic DNA were assessed using the NanoDrop UV/Vis spectrophotometer (Thermo Scientific, Wilmington, United States). The A260/A280 ratio was used to assess DNA purity and concentration, and the absorbance ratios of all samples ranged from 1.8 to 2.0. The genomic DNA extracts were stored at −20 °C until further use.

### 2.4 Internal transcribed spacer sequence analysis and primer/probe designs

A total of six internal transcribed spacer (ITS) sequences were acquired from GenBank (National Center for Biotechnology Information, www.ncbi.nlm.nih.gov/) to assist with the identification of species-specific single-nucleotide polymorphism (SNP) loci and to provide sequence alignment support for the primer and probe designs. The DNA sequences were aligned using BioEdit to find specific conserved sites for species identification and to identify intraspecific variations (Table 2). The primers and TaqMan probes were self-designed by our research group. The hydrolysis probes were labeled with FAM (fluorescein) at the 5′ end and minor groove binder (MGB) quencher unit at the 3′ end (Table 3).

**TABLE 2:** Internal transcribed spacer sequences downloaded from GenBank.

Species	GenBank number
*Fritillaria thunbergii* Miq. (ZBM)	MH588429.1, MF083549.1, MN184770.1
*Fritillaria hupehensis* (HBBM)	MN184759.1, MN184755.1, MN153742.1

**TABLE 3:** Primer and probe sequences of HBBM.

Target region	Primers and probe	Sequence (5′→ 3′)	Size of amplicon
ITS	Forward	5′-CAA​GTT​GCG​CCC​GAG​G-3′	47 bp
	Reverse	5′-TGA​CGC​CCA​GGC​AGG-3′	
	Probe	FAM-CTTTCGGTCGAGGGC-MGB	

### 2.5 qPCR assay development

The annealing temperatures used to determine the optimal temperature (or range) for the real-time fluorescent qPCR assay varied from 60 °C to 66 °C in steps of 1 °C. The qPCR products were visualized using the N1 high sensitivity cartridge kit and fully automatic nucleic acid analysis system (Qsep100, BIOptic, China). The 11 genomic DNA samples extracted from ZBM (ZBM-1 to ZBM-10 and ZBM-SD) and six adulterant HBBM samples (HBBM-1 to HBBM-5 and HBBM-SD) were selected as the DNA templates for qPCR amplification to validate the specificities of the probe and primers. A no-template control (NTC) comprising sterile distilled water instead of the DNA template was included in each qPCR run as a negative control to exclude the possibility of reagent contamination or non-specific amplification.

### 2.6 qPCR validation (specificity, sensitivity, and repeatability)

Six series of ten-fold dilutions of HBBM-SD DNA extracts ranging from 10 to 0.0001 ng/μL were used to construct the qPCR standard curves and determine the amplification efficiency. The standard curve corresponding to Ct for each DNA dilution was constructed using GraphPad Prism (v6.0) software. The linear correlation between the Ct value and initial concentration of the standard sample is given as Ct = a log[c] + b, where a is the slope and b is the intercept. Subsequently, the amplification efficiency was calculated from the slope of the standard curve as PCR efficiency = 10^–1^/slope^–1^. The amplification efficiency indicates the performance of the qPCR assay and ensures that the primer-probe system is within the acceptable range (90%–110%) for accurate quantification. Sensitivity was evaluated through the limit of detection (LOD), which was defined as the minimum DNA concentration that could be detected as a fluorescent signal. Three replicate DNA samples were extracted from the standard medicinal sample (HBBM-SD) to verify the biological replicates. Under identical qPCR conditions, each DNA sample was assayed in triplicate to determine the Ct values, and the mean Ct value of each group was calculated. The genomic DNA concentration of 0.1 ng/μL of HBBM (HBBM-SD) was selected to verify the technical replicates. Under identical qPCR conditions, three independent measurements were obtained from the samples in triplicate. The coefficient of variation was calculated as CV = (standard deviation/mean) to evaluate the repeatability of the experimental method.

### 2.7 Establishment of standard-curve-based quantitative method

Approximately 100 mg of the binary mixture powder of the standard medicinal materials (ZBM-SD and HBBM-SD) was mixed at each of six different percentages, namely 0% (0/100), 1% (1/99), 5% (5/95), 25% (25/75), 50% (50/50), and 100% (100/0). Then, 20 mg of each binary mixture sample was used to extract the DNA template, and 1.1 μL of the genomic DNA template was used for real-time fluorescence qPCR. Next, the standard curve was constructed using the logarithm of the proportion of HBBM adulteration in the ZBM sample as the abscissa (x-axis) and the corresponding amplification Ct value as the ordinate (y-axis) for quantitative detection of the adulteration ratio. The amounts of HBBM in the binary mixtures were deduced from the corresponding qPCR standard curves. To assess the accuracy of the quantification method, binary mixtures of HBBM and ZBM at predefined ratios of 50%, 25%, 5%, and 1% were analyzed, and pure HBBM (100%) was used for normalization. Here, ΔCt is defined as the difference between the Ct values of the pure HBBM standard and samples with different proportions of HBBM in the mixture with ZBM. The accuracy was evaluated using the recovery rate as follows:

ΔCt = Ct (100% ratio of HBBM) – Ct (other predefined mixture ratios).

Measured value = 1/2^−ΔΔCt^ × 100%

Recovery rate (%) = (measured value/theoretical value) × 100%

The above formula is only applicable when (100% ratio of HBBM) – Ct (other predefined mixture ratios).

## 3 Results

### 3.1 ITS sequence analysis and primer/probe designs

**FIGURE 2:**
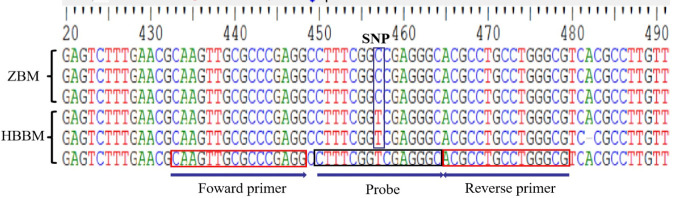
Design of probe and primers for detecting *Fritillaria hupehensis* (HBBM). The locations of the probe and primers on the ITS sequence are shown.

The conservation of the specific SNPs for ZBM and HBBM were determined by phylogenetic trees (neighbor-joining tree) generated from the downloaded ITS sequences (Figure 1B). Cluster analysis was then performed using the bootstrap method with 1,000 replicates to test the support value of each branch. We noted that ZBM and HBBM formed two distinct and well-supported clades with high bootstrap values; this demonstrates that the ITS region can be used to effectively and accurately distinguish ZBM from HBBM. After alignment, a specific SNP site was identified in the ITS sequence; based on this SNP site, the probe and primers were designed to detect HBBM (Figure 2). These were located within the ITS region from 432 to 479, with the length of the amplicon being 47 bp (Table 3).

### 3.2 qPCR assay optimization

**FIGURE 3:**
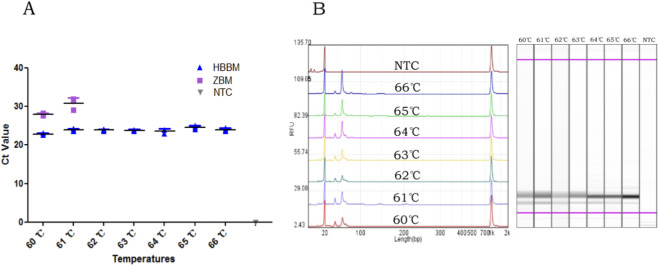
Optimization of annealing temperature for the proposed qPCR method: **(A)** Ct values at different annealing temperatures; **(B)** analysis of qPCR products of HBBM by the Qsep100 device.

The genomic DNA of HBBM could be successfully amplified in the temperature range of 60 °C–66 °C with Ct values ranging from 22.72 to 24.17. However, non-specific amplification targeting ZBM was observed at annealing temperatures of 60 °C–61 °C. When the annealing temperature was increased to 62 °C, non-specific amplification was no longer observed, indicating the satisfactory specificity of this method at this temperature. Additionally, even at a high annealing temperature of 66 °C, the Ct value did not change significantly compared to that at 62 °C (Figure 3A). The qPCR products of HBBM at different annealing temperatures were observed to remain at the same position and had the expected lengths (Figure 3B). Based on these results, 66 °C was determined to be the optimal annealing temperature for the proposed method.

### 3.3 qPCR validation

**FIGURE 4:**
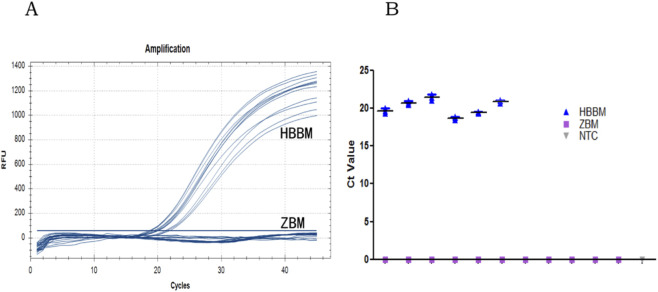
Specificity of the primers and probe for qPCR: **(A)** amplification curves of the qPCR assay; **(B)** Ct values of the HBBM and ZBM samples.

Samples from a total of 17 batches from diverse sources were amplified using the self-designed primers and probes. Of these, the six batches of HBBM samples showed relatively uniform amplification, with Ct values ranging from 18.77 to 21.82 (Figure 4A). Conversely, no amplification signals were observed in the 10 batches of ZBM samples and negative control (Figure 4B), demonstrating the excellent specificity of our method.

**FIGURE 5:**
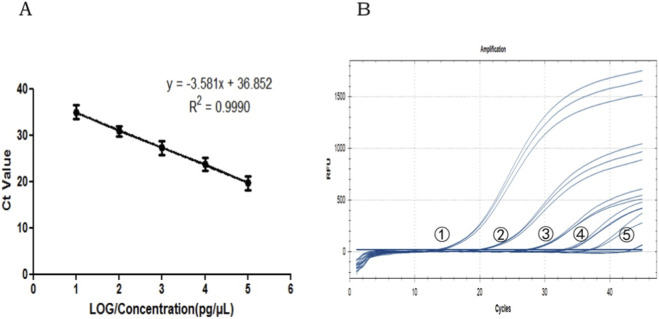
Amplification efficiency and sensitivity tests: **(A)** standard curve of the qPCR assay; **(B)** sensitivity of the qPCR assay. Lines ①–⑤ indicate DNA concentrations of 10, 1.0, 1.0 × 10^−1^, 1.0 × 10^−2^, and 1.0 × 10^−3^, respectively.

Genomic DNA concentrations ranging from 10 to 1.0 × 10^−4^ ng/μL were tested under the optimal PCR conditions, and the corresponding Ct values were found to range from 19.71 to 35.68. The amplification efficiency was calculated as 90.2% (slope = −3.581, R^2^ = 0.9993) (Figure 5A). The qPCR assay showed amplification when the genomic DNA of the HBBM samples was diluted from 10 to 1.0 × 10^−3^ ng/μL. However, no amplification signals were noted when the genomic DNA was further diluted to a concentration of 1.0 × 10^−4^ ng/μL. Thus, the LOD for our method was 1.0 × 10^−3^ ng/μL (Figure 5B). To assess the repeatability of the method, we performed triplicate measurements on the DNA extracts of the biological replicates and obtained a mean Ct value of 20.06 with a CV of 0.52%; then, we performed three independent tests on the technical replicates at a DNA concentration of 0.1 ng/μL and obtained a mean Ct value of 27.38 with a CV of 0.06%. These results demonstrate the reproducibility of the proposed method (Table 4).

**TABLE 4:** Repeatability test results (n = 6).

Repeatability	Ct (mean ± SD)	Average Ct	CV
Biological replicates	19.54 ± 0.31	20.06	1.03%
	20.59 ± 0.12		
	20.06 ± 0.13		
Technical replicates	27.35 ± 0.15	27.42	0.61%
	27.47 ± 0.17		
	27.44 ± 0.18		

### 3.4 Quantitative method validation

**FIGURE 6:**
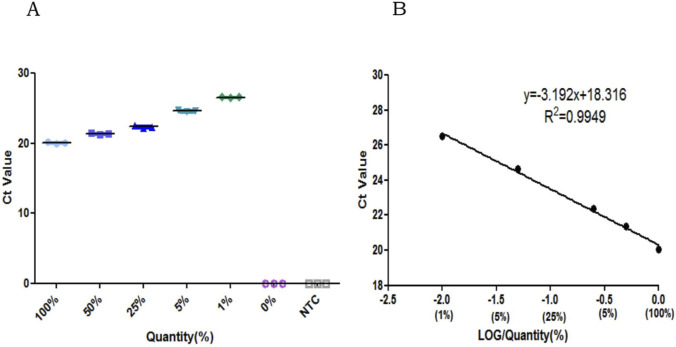
Quantification performance of the qPCR assay: **(A)** Ct values of the qPCR assay corresponding to different percentages of HBBM (0%, 1%, 5%, 25%, 50%, and 100%) added to a mixture of ZBM sample; **(B)** standard curve between the amount of HBBM adulteration in the ZBM sample (1%, 5%, 25%, 50%, and 100%) and Ct value.

The binary mixtures containing 50%, 25%, 5%, and 1% of the HBBM component were evaluated next; here, pure HBBM (100%) was set as the positive control while pure ZBM was set as the negative control. The Ct values for the mixed samples containing 100%, 50%, 25%, 5%, and 1% of HBBM exhibited a progressively increasing trend (Figure 6A). The equation of the standard curve was determined to be y = −3.192x + 18.316 with R^2^ = 0.9949; this indicates that there is a good linear relationship between the amount of HBBM adulteration in the ZBM sample (ranging from 1% to 100%) and Ct value (Figure 6B). The recovery rates for the quantification of the standardized mixture samples at ratios of 50%, 25%, 5%, and 1% were calculated to be 91.04%, 81.60%, 85.38%, and 113.85%, respectively (Table 5); these data demonstrate the satisfactory accuracy of quantification for the wide range of HBBM percentages (United States Pharmacopeial Convention, 2022).

**TABLE 5:** Recovery rates of the standardized mixture samples of HBBM in ZBM.

Theoretical value of HBBM (%)	Ct value (mean ± SD)	Measured value (%)	Recovery rate (%)	Recovery standard deviation (%)
100	20.08 ± 0.08	-	-	-
50	21.21 ± 0.10	40.52	81.04	8.85
25	22.37 ± 0.01	20.40	85.87	7.32
5	24.63 ± 0.19	4.27	89.22	6.78
1	26.54 ± 0.07	1.14	113.85	4.67

## 4 Discussion

Authenticating the species origins of TCM materials has long been a challenging issue. It is usually very difficult to distinguish adulterants from closely-related species in mixtures of real TCM, especially when the samples have lost their original characteristics, such as in the case of decoction pieces. Compared to traditional microscopic and physicochemical methods, DNA-based identification assays can distinguish confusing components in TCMs more definitely as hereditary substances are the cause and foundation of species differentiation. The qPCR assay exhibits higher specificity and sensitivity than general PCR assays while affording real-time monitoring and quantification capabilities (Kesmen et al., 2009). The key to achieving quality control in TCMs entails both qualitative and quantitative evaluations. Therefore, the qPCR approach may be the better choice. Our research group has long been committed to using the TaqMan probe-based qPCR detection method for TCM identification as it demonstrates high specificity, sensitivity, repeatability, and accuracy (Tao et al., 2025; Duan et al., 2026).

ITS sequences feature more variables and informative sites between different species and are already recognized as perfect molecular markers for identifying botanical drugs (Chen et al., 2010; Koo et al., 2019; Parvathy, et al., 2015; Schadock et al., 2015). Sequence comparisons showed that the ITS regions of ZBM and HBBM share 99.5% homology and that there are only a few SNP sites across the ITS sequences of both plants. One such SNP site within the ITS region was considered in this study to identify HBBM from ZBM using TaqMan probe qPCR techniques. We designed the MGB probe to specifically target HBBM, which largely enhanced the discriminative ability between different species and their amplification efficiencies. In this study, the qPCR assay features an amplicon of only 47 bp to specifically delineate ZBM from HBBM and quantify HBBM adulteration in a binary mixture with ZBM. Although the 47 bp short amplicon used in this study was specifically designed to ensure efficient DNA amplification in dried decoction pieces, we recognize that deeply processed products, such as those undergoing high-temperature boiling, honey-frying, or pill preparation, have not been included in this study. Nevertheless, further validation is required to confirm the performance of the proposed approach for deeply processed products. Future research efforts could explore the feasibility of further shortening the amplicon length to adapt to these complex samples.

Aside from the advantages of quantitative real-time PCR, there remain challenges and limitations to detecting and quantifying adulteration in TCMs. First, the purity of the template DNA is highly influenced by different extraction methods, which could compromise the accuracy of the quantitative results. Second, discrepancies in amplification efficiencies between the reference standards and test samples may cause biased quantification (Wang et al., 2014). Thus, we adopted an absolute quantification strategy based on a standard curve using standard reference medicinal materials with known adulteration ratios to establish a quantitative relationship, which allows normalization without relying on reference genes. However, the relatively large deviations in recovery rates for low-proportion adulteration samples (e.g., 1%) observed in this study may be attributed to factors such as DNA extraction homogeneity, random deviations in PCR amplification, and uniformity of the powder mixture. Thus, further in-depth research is required to enhance the application of qPCR in detecting adulteration in TCMs. This includes optimizing the DNA extraction methods, selecting appropriate reference materials, establishing rational quantification approaches, and adopting standardized analytical protocols (e.g., following the MIQE guidelines) to improve the assay accuracy and reproducibility (Bustin et al., 2024; Taylor and Mrkusich, 2014).

## 5 Conclusion

In this study, we developed a qPCR method for identifying and quantifying the adulterant HBBM in a mixture with ZBM with high specificity and accuracy. The proposed approach could be a useful tool for identifying HBBM adulteration in ZBM while providing a reference for qualitative and quantitative detection of other TCMs. Despite the advantages observed, we acknowledge certain limitations, including lack of validation for deeply processed products and challenges in detecting extremely low levels of adulteration. These limitations highlight the need for further research efforts to optimize and expand the applicability of the TaqMan probe-based real-time qPCR method.

## Data Availability

The datasets presented in this study can be found in online repositories. The name of the repository and accession numbers can be found at: https://www.ncbi.nlm.nih.gov/genbank/ (MH588429.1, MF083549.1, MN184770.1, MN184759.1, MN184755.1, MN153742.1).
